# Understanding parental self-medication with antibiotics among parents of different nationalities: a cross-sectional study

**DOI:** 10.1186/s41256-021-00226-y

**Published:** 2021-10-25

**Authors:** Zhongliang Zhou, Dantong Zhao, Huarui Zhang, Chi Shen, Dan Cao, Guanping Liu, Liang Zhu, Yu Fang

**Affiliations:** 1 School of Public Policy and Administration, Xi’an Jiaotong University, Xi’an, China; 2 Xi’an Lianhu District Huoshaobei Clinic, Xi’an, China; 3 Department of Health Care Management and Medical Education, School of Military Preventive Medicine, Air Force Medical University, Xi’an, China; 4 School of Pharmacy, Health Science Center, Xi’an Jiaotong University, Xi’an, China

**Keywords:** Parental self-medication with antibiotics, Nationality, Knowledge, Attitudes, Practices

## Abstract

**Background:**

There is an increasing trend on the practices of parental self-medication with antibiotics (PSMA) around world, accelerating the antibiotic abuse. This study aims to examine the nationality differences in the practices of PSMA and knowledge, attitudes and practices (KAP) toward antibiotic use, and understand the practices of PSMA among parents of various nationalities in China.

**Methods:**

A cross-sectional study based on a structured questionnaire survey was conducted in Xi’an, Shaanxi Province, China, from September 2018 to October 2018. A total of 299 respondents participated in. The practices of PSMA (a dichotomous variable) and KAP toward antibiotic use (a continuous variable) served as dependent variables. Participant’s nationality was regarded as the independent variable. Binary logistic regression and ordinary least square regression were employed to examine the association between parent’s nationality and the practices of PSMA, and KAP toward antibiotic use, respectively.

**Results:**

121 (40.88%) Chinese, 100 (33.76%) other Asians and 75 (25.34%) Occidentals were included in final analysis, with a sample size of 296. Chinese were more likely to practice PSMA (OR = 7.070; 95% CI 1.315, 38.01), with worse knowledge (Coef. = − 0.549; 95% CI − 1.021, − 0.078), attitudes (Coef. = − 3.069; 95% CI − 4.182, − 1.956) and practices (Coef. = − 1.976; 95% CI − 3.162, − 0.790) toward antibiotic use, compared to their Occidental counterparts. The main reasons for the practices of PSMA were enough previous medication experience (80.49%) and same ailments with no need to see a doctor (39.02%), with common symptoms such as fever (60.98%) and cough (58.54%). Purchasing antibiotics at pharmacies (92.08%) and using leftover antibiotics (26.83%) were usual approaches.

**Conclusions:**

The study highlights the gaps in the practices of PSMA and KAP toward antibiotic use among parents of different nationalities. The access to obtain antibiotics from pharmacies reflects the pharmacists’ unaware of laws on prescription of antibiotics, fierce competition in the pharmacy market, and the government’s lax supervision in China. It suggests the need to improve pharmacists’ training, enforce current legislations on pharmacy market regarding the sale of antibiotics, and provide practical and effective educational interventions for residents about antibiotic use.

**Supplementary Information:**

The online version contains supplementary material available at 10.1186/s41256-021-00226-y.

## Background

Bacterial resistance, a main adverse effect of antibiotic use, is mainly driven by irrational and excessive use of antibiotic in both communities and clinical settings [1]. It is regarded as an important public health concern [2] and one of the biggest threats facing global health [3]. It not only increases disease morbidity and mortality, reduces the efficiency of disease treatment, increases the economic burden of patients, but also makes it easy to generate drug-resistance or multidrug-resistance bacteria to further harm health [4]. As the World Health Organization (WHO) points out, “a post-antibiotic era—in which common infections and minor injuries can kill everything—far from being an apocalyptic fantasy, is instead a very real possibility for the twenty-first century” [5]. It is great necessary to focus on irrational antibiotic use.

There is an increasing trend on practicing self-medication with antibiotics (SMA) in both developed and developing countries in recent years, which is one of the most dangerous and prevalent inappropriate antibiotic use behaviors [6]. A systematic review conducted in Netherlands reported that the prevalence of SMA ranged from 2% to 20% in Europe, with Sweden at 2%, Slovakia 3%, Romania 16% and Greece at 20% [7]. Another literature review showed that frequent practices of SMA varied from 23.6% to 85% in Asian countries, with 23.6% in Bhutan, 50% in Lebanon, 63% in Kazakhstan, 83.3% in Vietnam and 85% in Lao PDR [8]. Moreover, much higher rates of SMA were found in Africa, such as in Ghana (70%) [9], Kenya (79.9%) [10], and Nigeria (93.9%) [11]. Self-medication can be defined as the use of drugs with the aim to treat self-diagnosed disorders or symptoms, or the intermittent or continued use of a prescribed drug for chronic or recurrent disease and symptoms [12]. Antibiotics for self-medication are usually obtained without a prescription, or by resubmitting old prescriptions to purchase medicines at retail pharmacies, or sharing medicines with relatives or friends, or using leftover medicines stored at home [6, 13, 14].

In practice, SMA also includes the medication for family members, in particularly, the therapy for children or the old [12]. Previous studies presented that children and adolescents are the most widely users on antibiotics [15], whose medicines are usually purchased by parents themselves [16]. It is commonly known as parental self-medication with antibiotics (PSMA), accelerating the antibiotic abuse [17]. The practices of PSMA are also prevalent around the world, especially in low- and middle-income countries (LMICs) [18, 19]. It was reported that the prevalence of PSMA was 59.4% in China [20], 42.05% in Philippines [21], 43% in Uganda [22] and Nigeria [17], yet this proportion was as low as 22.7% in Greece [23].

Unlike high-income countries, non-prescribed antibiotics (NPA) (i.e., the use of leftover antibiotics, the use of antibiotics recommended by pharmacy staffs, sharing antibiotics with families or friends [24]) are easy to access in LMICs [18, 25], associated with the lack of robust mechanisms of health education and enforcement measures to control the unnecessary and unreasonable use of antibiotics [24, 26]. Moreover, insufficient and inconsistent knowledge and attitudes toward antibiotic use, high expectations on the curative effect of antibiotics, and poor quality of healthcare facilities, are factors driving inappropriate use of antibiotics [24]. Regarding the above gaps in the prevalence of PSMA around the world and various factors influencing the utilization of antibiotics, we therefore hypothesis that the practices of PSMA may differ among parents of different nationalities in China.

As we all know, the irrational or overuse of antibiotics is caused by either health service supplier or demander. The supply side factors mainly include the lack of supervision [27], corruption [28], physicians’ profit-seeking behavior [29], insufficient knowledge [30] and so on. The demand side reasons are probably due to the public’s inadequate knowledge, attitudes and practices (KAP) toward antibiotic use [31–38]. The actual difference among various populations cannot be identified unless controlling the same supplier or the same demander. However, few studies to our knowledge have examined such essential gap. According to the theory of Andersen’s Behavioral Model of Health Services Use, health seeking behavior can be influenced by health system environment [39, 40]. The public is regulated by unified health policies when residing in the same city. This study therefore selected parents of different nationalities living in the same city, on behalf of different health service demanders but the same supplier.

In the current study, we aim to (1) explore the association between parent’s nationality and the practices of PSMA; (2) examine the nationality differences in KAP toward antibiotic use; (3) understand the practices of PSMA among parents of various nationalities living in China.

## Methods

### Study design and setting

This was a cross-sectional study conducted in Xi’an Hi-Tech International School, International Department (XHISID), Shaanxi Province, China, for a period of 2 months from September 2018 to October 2018. XHISID was founded in 2003. It is the first International Baccalaureate (IB) World School authorized by the IB Organization in the northwest region of China, also a key international education program supported by the Hi-Tech Zone Administrative Committee of Xi’an, Shaanxi. XHISID has more than 300 students from about 15 countries around the world. It is a 15-year consistent school. School offers the IB-Primary Years Programme, IB-Middle Years Programme and the Diploma Programme continuum from kindergarten to high school. It is the only SAT and AP test center authorized by the American College Board in the northwestern region of China. XHISID provides a non-profit education focused on enabling confident, open-minded global citizens through life-long learning and a sense of community.

### Sampling

The sample size was calculated using Raosoft [41], an online sample size calculator frequently applied in studies [42–44]. We assumed the prevalence of PAMA to be 30% among various populations (referring to previous studies conducted in various regions) and determined the confidence interval at 95% with the margin of error 5%. The population size was assumed to be 20,000 as suggested by the Raosoft, since there was no official statistics on international populations in Xi’an and the sample size doesn't change much for populations larger than 20,000 [41]. The required sample size estimated for the survey was 318. A convenience sample of 350 participates were included in this study to account for a 10% non-response rate.

### Participants and data collection

The survey was conducted at classrooms after parents meeting. Participants were eligible to be included if they: (1) being a parent whose child was studying at XHISID; (2) having no hearing or visual disorder; (3) being literate; (4) volunteering to participate in. An introduction to study purpose, significance and privacy safety was informed to participants by our investigators before the investigation. Respondents were required to self-complete questionnaire with their informed consent. Finished questionnaires were collected by the investigators on the spot. A total of 299 individuals participated in the investigation and accomplished questionnaire.

### Development of questionnaire

The questionnaire was developed by reviewing applicable comparable studies [18, 33, 45–49] and consultation with pharmacy experts. Face and content validation of the questionnaire was performed by pharmacy professionals and academics. Feedback was collected to improve the questionnaire presentation, clarity, logicality and congruency of meaning. The questionnaire was originally developed in English (see Additional file 1), which was then translated into Chinese (see Additional file 2), for non-Chinese and Chinese participates, respectively. A pilot study was carried out to address any ambiguity in the questions and the acceptability to the participants among 30 respondents. The questionnaire consisted of three main parts: (1) the socio-economic characteristics of parents and children; (2) the practices of PSMA; (3) the assessment of KAP toward antibiotic use. Cronbach’s alpha was 0.85 for the part of KAP toward antibiotic use.

### Measurement

296 individuals were eventually analyzed excluding the missing values. PSMA served as a dependent variable in this study, measured by question, “Did you self-medicate your child with antibiotics in the past 6 months”, with an answer of “No” or “Yes”, coding 0 and 1. Another dependent variable was KAP toward antibiotic use. Antibiotic knowledge was composed of 6 questions, with an answer of “True”, “False” and “Not sure”. We classified the answer into “Wrong” (including incorrect answers and “Not sure”) and “Right” (correct answers), coding 1 and 2. The sum score ranged from 6 to 12, with higher score indicating better antibiotic knowledge. Antibiotic attitudes consisted of 14 items. Each item is answered on a five-point Likert scale (“Totally agree” to “Totally disagree”), coding from 1 to 5. A total scale score was computed by summing item scores, with a range of 5 to 25. Higher scores indicated better antibiotic attitudes. Practices of antibiotic use were measured by 12 items with a five-point Likert response (“Always” to “Never”), coding from 1 to 5. Item scores were summed and converted to a scale ranging from 6 to 30, with higher scores presenting better antibiotic behaviors. The total antibiotic score was the sum of the above scales, ranging from 17 to 67, where higher values indicated better antibiotic perceptions.

Parent’s nationality was an independent variable, with a category of Chinese, other Asian and Occidental. Control variables included parent’s gender (male, female; a dichotomous variable), age (27–52 years; a continuous variable), education level (below Bachelor, Bachelor, Master and above; a categorical variable) and income (< 0.5 million, 0.5–1 million, > 1 million; a categorical variable), having medical staff or not in family (no, yes; a dichotomous variable), child’s age (2–19 years; a continuous variable) and gender (male, female; a dichotomous variable), the only child or not (no, yes; a dichotomous variable) and having medical insurance or not (no, yes; a dichotomous variable). In addition, total antibiotic score (17–67; a continuous variable) was controlled when examining the association between parent’s nationality and the practices of PSMA.

### Statistical analysis

The data was entered into a database using the Epidata 3.1, and transferred to Stata statistical software (version15.0; StataCorp LP, College Station, Texas) for all analysis. One-way ANOVA and Kruskal–Wallis test were used to compare KAP toward antibiotic use among parents of different nationalities. The association between parent’s nationality and the practices of PSMA was examined using the binary logistic regression model adjusted for socio-economic characteristics of parents and children, and antibiotic score. Ordinary least square (OLS) regression controlling for socio-economic characteristics of parents and children was employed to examine the association between parent’s nationality and KAP toward antibiotic use. *P* value was at the significance level α = 0.05.

## Results

The response rate was 84.57%. As Table 1 is shown, 121 (40.88%) Chinese parents, 100 (33.78%) other Asian parents and 75 (25.34%) Occidental parents comprised the study sample. The proportion of female was 84.30%, 73.00% and 37.33% among parents of three nationalities, respectively. The average age of the respondents was 37.96 (SD = 5.45) years, 40.71 (SD = 3.63) years and 39.44 (SD = 4.63) years. Minority of the respondents’ families had medical staffs and majority of children had been covered by medical insurance.

**Table 1:** General characteristics of parents and children

Variables	Chinese (N = 121)		Other Asian (N = 100)		Occidental (N = 75)	
	N	%	N	%	N	%
*Parents*						
Gender						
Male	19	15.70	27	27.00	47	62.67
Female	102	84.30	73	73.00	28	37.33
Age, years						
Mean (SD)	37.96 (5.45)		40.71 (3.63)		39.44 (4.63)	
Education level						
Below bachelor	22	18.19	29	29.00	4	5.33
Bachelor	55	45.45	52	52.00	26	34.67
Master and above	44	36.36	19	19.00	45	60.00
Medical staff in family						
No	96	79.34	86	86.00	55	73.33
Yes	25	20.66	14	14.00	20	26.67
Income, million, CNY						
< 0.5	62	51.24	37	37.00	67	89.33
0.5–1	30	24.79	46	46.00	5	6.67
> 1	29	23.97	17	17.00	3	4.00
*Children*						
Gender						
Male	61	50.41	40	40.00	53	70.67
Female	60	49.59	60	60.00	22	29.33
Age, years						
Mean (SD)	7.46 (3.73)		10.07 (3.85)		8.89 (5.42)	
Medical insurance						
No	31	25.62	21	21.00	12	16.00
Yes	90	74.38	79	79.00	63	84.00

Assessed from full sample, N = 296

SD = standard deviation

It was found that 13.85% of the respondents practiced PSMA in the past 6 months. The prevalence of PSMA among Chinese was higher than that among other Asians and Occidentals (28.10%, 5.00% and 2.67% for Chinese, other Asians and Occidentals, respectively, *P* < 0.001).

As Table 2 is presented, respondents practiced an average of 1.44 (SD = 0.63) times PSMA in the past 6 months, with a duration of 3.46 (SD = 1.12) days. The main reasons for the practices of PSMA were enough previous medication experience (80.49%) and the same ailments with no need to see a doctor (39.02%), with the common symptoms such as fever (60.98%), cough (58.54%), bronchitis (43.90%), sore throat (34.15%) and cold (17.07%). Sources of antibiotic information were mainly previous medication experience (80.49%), suggestions from relatives and friends (21.95%) and recommendations of pharmacy staffs (19.51%). Purchasing antibiotics at pharmacies (92.08%) and using leftover antibiotics (26.83%) were usual approaches to access to antibiotics for PSMA.

**Table 2:** Description of the practices of parental self-medication with antibiotics (PSMA)

Variables	Mean/N	SD/%
*Medication*	*Mean*	*SD*
Frequency in the past 6 months, times	1.44	0.63
Duration, days	3.46	1.12
*Reasons (Multi-choice)*	*N*	*%*
Same ailments with no need to see a doctor	16	39.02
Enough previous medication experience	33	80.49
Long waiting time in the clinics	4	9.76
Convenience	1	2.44
Expensive consultation fees	1	2.44
*Symptoms (Multi-choice)*	*N*	*%*
Cold (Runny nose or nasal congestion)	7	17.07
Cough	24	58.54
Fever	25	60.98
Sore throat	14	34.15
Bronchitis	18	43.90
Body ache	3	7.32
Headache	3	7.32
Emesis	0	0.00
Diarrhea	2	4.88
Otitis media	4	9.76
Skin trauma	0	0.00
*Sources of antibiotic information (Multi-choice)*	*N*	*%*
Previous medication experience	33	80.49
Suggestions from relatives and friends	9	21.95
Recommendations of pharmacy staffs	8	19.51
Internet knowledge	2	4.88
Drug instruction	5	12.20
*Approaches (Multi-choice)*	*N*	*%*
Leftover of previous antibiotics	11	26.83
Purchasing antibiotics at pharmacies	38	92.08
Given by others	1	2.44

Assessed from individuals with the practices of PSMA, N = 41

SD = standard deviation

KAP toward antibiotic use among parents of different nationalities are showed in Table 3. Occidental parents performed better than their Chinese counterparts, followed by other Asians (10.07 vs. 9.65 vs. 8.98 regarding antibiotic knowledge, 20.53 vs. 18.46 vs. 17.02 regarding antibiotic attitudes, 28.23 vs. 26.07 vs. 22.80 regarding antibiotic behaviors among Occidentals, Chinese and other Asians, respectively. *P* < 0.001). With regard to the perception of antibiotic use, Occidental parents still performed better than Chinese, followed by other Asians (58.83 vs. 54.18 vs. 48.80, *P* < 0.001).

**Table 3:** knowledge, attitudes and practices (KAP) toward antibiotic use among parents of different nationalities

Variables	Chinese (N = 121)		Other Asian (N = 100)		Occidental (N = 75)		*F/χ*^2^	*P* value
	Mean	SD	Mean	SD	Mean	SD		
Knowledge score^a^	9.65	1.55	8.98	1.29	10.07	1.49	12.77	< 0.001
Attitude score^a^	18.46	3.54	17.02	3.42	20.53	3.74	20.96	< 0.001
Practice score^b^	26.07	4.03	22.80	3.99	28.23	2.30	85.00	< 0.001
Antibiotic score^b^	54.18	7.32	48.80	6.80	58.83	6.18	75.32	< 0.001

Antibiotic score was computed by summing antibiotic knowledge, attitude and practice score

Assessed from full sample, N = 296

SD = standard deviation

^a^One-way ANOVA

^b^Kruskal–Wallis test

The results of regression analyses examining the association between parent’s nationality and the practices of PSMA, and the association between nationality and KAP toward antibiotic use are conducted and presented in Table 4. It was found that Chinese were more likely to practice PSMA (OR = 7.070; 95% CI 1.315, 38.01), and performed worse in knowledge (Coef. = − 0.549; 95% CI − 1.021, − 0.078), attitudes (Coef. = − 3.069; 95% CI − 4.182, − 1.956) and practices (Coef. = − 1.976; 95% CI − 3.162, − 0.790) toward antibiotic use, compared to their Occidental counterparts.

**Table 4:** The association between parent’s nationality and 1) the practices of PSMA, 2) knowledge, attitudes, and practices toward antibiotic use

Variables	PSMA^a^	Knowledge^b^	Attitudes^b^	Practices^b^
	OR	Coef	Coef	Coef
	(95% CI)	(95% CI)	(95% CI)	(95% CI)
Nationality of parents				
Occidental	Ref			
Chinese	7.070*	− 0.549*	− 3.069***	− 1.976**
	(1.315–38.010)	(− 1.021– − 0.078)	(− 4.182– − 1.956)	(− 3.162– − 0.790)
Other Asian	0.500	− 0.974***	− 4.088***	− 4.992***
	(0.059–4.266)	(− 1.484– − 0.463)	(− 5.294– − 2.882)	(− 6.276– − 3.707)
Gender of parents				
Male	Ref			
Female	1.191	− 0.110	2.908***	0.991*
	(0.339–4.183)	(− 0.502–0.281)	(1.983–3.833)	(0.005–1.977)
Age of parents, years	0.971	− 0.067**	− 0.066	− 0.170**
	(0.874–1.079)	(− 0.110– − 0.024)	(− 0.168–0.036)	(− 0.279− − 0.061)
Education level of parents				
Below bachelor	Ref			
Bachelor	0.611	− 0.478*	0.528	1.296*
	(0.209–1.788)	(− 0.932– − 0.024)	(− 0.544–1.600)	(0.153–2.438)
Master and above	0.495	− 0.259	0.529	2.265***
	(0.148–1.656)	(− 0.749–0.230)	(− 0.627–1.685)	(1.033–3.496)
Income, million, CNY				
< 0.5	Ref			
0.5–1	3.568*	− 0.028	− 0.685	− 0.199
	(1.312–9.702)	(− 0.434–0.378)	(− 1.644–0.275)	(− 1.222–0.823)
> 1	0.661	0.304	− 0.736	0.251
	(0.201–2.178)	(− 0.173–0.782)	(− 1.865–0.392)	(− 0.951–1.453)
Having medical staff or not in family				
No	Ref			
Yes	2.764*	0.717***	0.558	0.636
	(1.080–7.077)	(0.307–1.126)	(− 0.409–1.526)	(− 0.395–1.667)
Gender of children				
Male	Ref			
Female	0.734	0.145	0.131	− 0.511
	(0.318–1.697)	(− 0.185–0.476)	(− 0.648–0.911)	(− 1.342–0.320)
Age of children, years	0.856*	− 0.041	− 0.098	0.0521
	(0.742–0.988)	(− 0.087–0.010)	(− 0.206–0.010)	(− 0.063–0.167)
The only child or not				
No	Ref			
Yes	1.042	− 0.219	− 1.331**	− 1.732***
	(0.456–2.383)	(− 0.570–0.132)	(− 2.159– − 0.502)	(− 2.615– − 0.849)
Having medical insurance or not				
No	Ref			
Yes	0.364*	− 0.236	− 0.103	− 0.476
	(0.141–0.943)	(− 0.638–0.167)	(− 1.053–0.848)	(− 1.489–0.536)
Antibiotic score	0.923*			
	(0.865–0.984)			

Assessed from full sample, N = 296

Antibiotic score was computed by summing antibiotic knowledge, attitude and practice score

OR = odds ratio, Coef. = coefficient, 95% CI = 95% confidence interval

^a^Binary logistic regression

^b^Ordinary least square regression

***P < 0.001, **P < 0.01, *P < 0.05

## Discussion

To our knowledge, this is the first study to understand the practices of PSMA, and KAP toward antibiotic use among parents of various nationalities under Chinese health system. Our results demonstrated that: (1) Chinese were more likely to practice PSMA, and performed worse KAP toward antibiotic use, compared to their Occidental counterparts; (2) the practices of PSMA occurred for symptoms such as fever and cough, mainly because of previous medication experience and the same ailments with no need to see a doctor; (3) the common sources of antibiotic information included previous medication experience, and the recommendations from relatives, friends and pharmacy personnel; (4) antibiotics for self-medication were often obtained from pharmacies and leftover of previous medication.

It is well known that unnecessary and irrational use of antibiotics is a major cause of bacterial resistance [50], which is accelerated by the prevalent fact of PSMA. Previous studies showed that the rate of the practices of PSMA was high in China [51], ranging from 32.20% to 62% [18, 20, 48, 52]. However, it was only 4.0% in urban U.S., 12.1% in suburban U.S. [53], and 22.7% in the Greece [23]. As for other Asian countries, it was 43.9%, 39.2% and 29.8% in Saudi Arabia [54], Jordan [55] and Vietnam [56], respectively. It’s worth noting that nationality was associated with the practices of PSMA in this study, showing that Chinese parents were more likely to self-medicate with antibiotics for children than Occidental parents, the same as the above findings conducted in separate countries [18, 20, 23, 48, 52, 53]. However, the essential difference in the practices of PSMA among populations of various nationalities is revealed in this study, as the legislations and healthcare services provider were unbiased for residents (under Chinese health system), which makes it more convincing than directly comparing the practices of PSMA among various countries or regions.

The practices of PSMA difference among parents of various nationalities could be explained by KAP toward antibiotic use that parents performed. The finding outlined significant gap in KAP among parents of different nationalities, indicating that Occidental parents performed better than Chinese, which is less confounded by healthcare provider factors as they lived in the same city under the same health system. Several studies state that the control of irrational use of antibiotics is mainly dominated by regulation of physician prescription and correct KAP toward antibiotic use [57, 58]. Plenty of campaigns educating the public about the appropriate use of antibiotics were successfully held at a national or regional level in many high-income countries from 1990 to 2007, including Australia, Canada, France, the UK, New Zealand and so on [59]. China has also demonstrated a series of educational activities on rational use of antibiotics since 2010 [60]. Considering educational campaigns about the appropriate use of antibiotics in European and American countries were carried out earlier than China and have already got excellent achievements [59], Occidental parents naturally performed much better KAP toward antibiotic use. It suggests that intensive educational interventions that provide more and accurate knowledge about the rational use of antibiotics for residents at community level are urgently needed in China.

Regarding the antibiotics for self-medication, previous studies showed that they were usually obtained from over-the-counter purchases at retail pharmacies and leftover medications from previous prescriptions [61, 62], in line with our findings. Actually, despite being prescription-only medicines (POM), antibiotics are available without a valid prescription in many LMICs [26], with an estimation of over 50% [6]. In China, a recent study conducted in 13 provinces indicated that NPA could be purchased at 83.6% pharmacies, and only 11.9% pharmacies asked the consumers if they had a prescription [62]. This raises concern about the role of pharmacies in the misuse of antibiotics, given that the Chinese government has officially banned non-prescription dispensing of antibiotics since 2004 [63]. It reveals the fierce competition in the pharmacy market, the poor inspection of pharmacies, the fragile law enforcement capabilities, and the pharmacists’ unaware of local laws and regulations on prescription of antibiotics [64, 65]. Moreover, what the individuals practicing PSMA may not realize is that using NPA exposes their children to various health risks, increasing the healthcare costs and the burden of resistant infections [66]. Therefore, educational interventions should target not only residents at community level, but also pharmacists in pharmacies at society level [67], combined with a series of strategies to improve pharmacists’ training, enforce current legislations focusing on frequent supervisions, inspections and penalties of all pharmacies in China.

Nowadays, driven by the fact of aggressive marketing strategies and high expectations for antibiotics, antibiotics are often mistaken used to treat self-diagnosed non-bacterial diseases and viral infections. Children suffering from the Upper Respiratory Tract Infection (URI) with symptoms of fever, cold, cough and sore throat are more likely to be self-medicated with antibiotics [68–70]. Furthermore, the evidences and our findings indicate that the practices of PSMA are attributable to the same ailments with no need to see a doctor [46], the long waiting time in the clinics [71], storing antibiotics at home [48] and enough previous medication experience [72]. Therefore, healthcare providers need to take measures to provide convenience for the patients and reduce the waiting time at healthcare facilities as far as possible, thus enabling the access to healthcare facilities and the guidance of wise and rational use of antibiotics.

### Limitations

We acknowledge several limitations of this study that need to be addressed in future studies. Firstly, this study is limited in the sample population conducted in an International School, resulting in inadequate sample size. Participates are much richer and better educated than general populations because of the convenience sampling, which leads to the selection bias and cannot be well generalized to the whole population of different nationalities. Secondly, self-administered questionnaire can be subject to social-desirability bias, leading to untrue feelings and practices. Thirdly, participates’ medical insurances are not identified in this study. Different type of medical insurance coverage may lead to different health seeking behavior and the practices of PSMA. Fourthly, local Chinese may be more familiar with health system and easier to get access to antibiotics compared to non-Chinese, although they both live in the same city for a long time. Fifthly, the lack information of grandparents of the children may underestimate the practices of PSMA, since grandparents are also the main caregivers for children [73]. In addition, no data are available to understand the practices of PSMA among African participates in this study, thus the findings are incomplete and defective. Considering the high prevalence of SMA [9–11, 66] and the scarce literatures on the practices of PSMA in Africa [17, 22], future study understanding the practices of PSMA and KAP toward antibiotic use among African are need. Therefore, the findings in this study need to be cautiously explained and extrapolated. Despite the aforementioned limitations, findings from the current study supplement and improve the existing literatures on the practices of PSMA and KAP toward antibiotic use, against the background of different demanders but the same health services supplier. This study provides crucial empirical evidence regarding the nationality differences in the practices of PSMA and KAP toward antibiotic use, with practical implications for the improvement of regulations and the promotion of health education on antibiotics for the public in China.

## Conclusions

The study highlights the gaps in the practices of PSMA and KAP toward antibiotic use among parents of various nationalities, indicating that Chinese are more likely to practice PSMA, with worse KAP toward antibiotic use, compared to their Occidental counterparts. The access to obtain antibiotics from pharmacies reflects the pharmacists’ unaware of laws and regulations on prescription of antibiotics, fierce competition in the pharmacy market and the government’s lax supervision in China. These findings suggest the need for Chinese health sector and government not only to strengthen the supervision and regulation on pharmacy market regarding the sale of antibiotics, but also improve pharmacists’ training, and provide practical and effective educational interventions for the public about rational use of antibiotics via doctor-patient communication, brochures, poster, lectures and Internet, to discourage the practices of PSMA.

## Supplementary Information

**Additional file 1:** Questionnaire of parental self-medication with antibiotics among parents of different nationalities (English Version).

**Additional file 2:** Questionnaire of parental self-medication with antibiotics among parents of different nationalities (Chinese Version).

## Data Availability

The datasets used and/or analyzed during the current study are available from the corresponding author on reasonable request.

## Abbreviations

WHO: World Health Organization
NPA: Non-prescribed antibiotics
SMA: Self-medication with antibiotics
PSMA: Parental self-medication with antibiotics
KAP: Knowledge, attitudes and practices
LMICs: Low- and middle- income countries
XHISID: Xi’an Hi-Tech International School, International Department
IB: International Baccalaureate
OLS: Ordinary least square
POM: Prescription-only medicines
URI: Upper Respiratory Tract Infection
