# Supplementary material for: Understanding parental self-medication with antibiotics among parents of different nationalities: a cross-sectional study
Source: Glob Health Res Policy. 2021 Oct 25;6:42. doi: 10.1186/s41256-021-00226-y (PMC8543833; doi:10.1186/s41256-021-00226-y)
Supplement: Supplementary file 1 — Additional file 1: Questionnaire of parental self-medication with antibiotics among parents of different nationalities (English Version). [file 41256_2021_226_MOESM1_ESM.pdf]

Additional file 1

Questionnaire of parental self-medication with antibiotics among parents of different nationalities

Dear participates,

Considering the prevalent fact that children and adolescents are easily self-medicated with antibiotics by their parents around the world, and different perceptions and habits of antibiotic use between Chinese and non-Chinese parents, the **PURPOSE** of this study is to explore the status quo of parental self-medication with antibiotics among parents of different nationalities, and parents' knowledge, attitudes and practices toward antibiotic use. The findings will contribute to the understanding of parental self-medication with antibiotics and the improvement of related regulations and health educations.

Filling out the questionnaire is completely **VOLUNTARY** and **ANONYMOUS**. The survey is only used for academic research. It will take you **3-5** minutes to complete the questionnaire.

Thanks for your understanding and support!

✎ Glossary

1. Antibiotics: *are drugs that fight bacteria. Antibiotics do not work against viruses, such as those that cause colds and flu. Common antibiotics include penicillin, amoxicillin, cefazolin (Pioneer Ⅴ), cefradine, cefuroxime, cefixime, celecoxib, roxithromycin, azithromycin and so on.*

2. Upper Respiratory Tract Infections (URIs): *is defined as self-limited irritation and swelling of the upper airways with associated cough, but occasionally the viral infection spreads to adjacent organs, resulting in different clinical manifestations. URIs involve the nose, sinuses, pharynx, larynx, and large airways. The main symptoms are nasal congestion, runny nose, sneezing, sore throat, coughs, low-grade fever, headache. The occurrence of URIs displays a clear seasonal variation.*

3. Self-medication: *is defined as the use of drugs with the aim to treat self-diagnosed disorders or symptoms, or the intermittent or continued use of a prescribed drug for chronic or recurrent disease and symptoms.*

✎ Instruction

Please type "√ " in the "□" of the corresponding option, or fill out the content directly when you encounter "_______".

(Only one option can be selected for each item unless specified.)

Part I: Demographic backgrounds

A. Parents:

1. Gender:
   □ Male □ Female

2. Age: ____ years

3. Nationality:
   □ Chinese □ Other Asian □ European or American

4. You are the child's:
   □ Parent □ Grandparent □ Others (please specify)

5. Education:
   □ Master or above □ Bachelor □ Below Bachelor

6. Your occupation
   □ Staff □ Cadres □ Professionals and technicians
   □ Famer □ Businessman □ Self-employed □ Others (please specify)

7. Are there medical workers in your family:
   □ Yes □ No

8. Annual household income: _______(million)

B. Children:

9. Gender:
   □ Male □ Female

10. Age: _____ years

11. Nationality:
    □ Chinese □ Other Asian □ European or American

12. Grade:
    □ Kindergarten □ Primary School □ Middle School □ High School

13. Single child:
    □ Yes □ No

14. Does your child have medical insurance (including Urban Resident Basic Medical Insurance and

commercial insurance):
□ Yes □ No

Part II Parental self-medication with antibiotics

15. Did you **self-medicate with antibiotics for your child** in the past six months?
(Note: Continuing to answer the question 15 if you select "Yes"; Skipping to the question 21 in Part III if you select "No".)
□ Yes □ No

16. In the past six months you have self-medicated with antibiotics for your child ________ **times**; the **name** of the antibiotic used is ____________________; the medication lasted for _____ **days**.

17. The **reasons** for your self-medication with antibiotics for your child are: ***(Multi-choice)***
□ Same ailments with no need to see a doctor □ Enough previous medication experience
□ Long waiting time in the clinics □ Convenience □ Expensive consultation fees
□ Others _______ (please specify)

18. The **symptoms** when you self-medicate with antibiotics for your child are: ***(Multi-choice)***
□ Runny nose □ Nasal congestion □ Cough □ Fever □ Sore throat □ Bronchitis
□ Body ache □ Headache □ Emesis □ Diarrhea □ Otitis media □ Skin trauma
□ Others _______ (Please specify)

19. The **sources of antibiotic information** are: ***(Multi-choice)***
□ Previous medication experience □ Suggestions from relatives and friends
□ Recommendations of pharmacy staffs □ Internet knowledge
□ Drug instruction
□ Others _______ (Please specify)

20. The **approaches** to obtain antibiotics are: ***(Multi-choice)***
□ Leftover of previous antibiotics
□ Purchasing antibiotics at pharmacies
□ Given by others
□ Others _______ (Please specify)

Part III: Knowledge, attitudes and practices toward antibiotic use

A. Knowledge

How about your medical knowledge about antibiotics? Please answer the following question.

21. Antibiotics are **anti-inflammatory drugs**.
□ True □ False □ Not sure

22. Antibiotics can **fight viruses**.
 □ True □ False □ Not sure

23. Antibiotics should be purchased by **prescription** at pharmacies.
 □ True □ False □ Not sure

24. Most of the Upper Respiratory Tract Infection are **viral infection**.
 □ True □ False □ Not sure

25. **Repeatedly using an antibiotic** is prone to have bacterial resistance.
 □ True □ False □ Not sure

26. **Insufficient dosage** of antibiotics leads to bacterial resistance.
 □ True □ False □ Not sure

B. Attitudes

What's your attitudes about antibiotic use when your child is ill? Please answer the following questions.

27. Do you agree that antibiotics should be used immediately when your child has **Upper Respiratory Tract Infection**?
 □ Totally agree □ Agree □ Fair □ Disagree □Totally disagree

28. Do you agree that **expensive** antibiotics **work better** with **fewer adverse effects**?
 □ Totally agree □ Agree □ Fair □ Disagree □Totally disagree

29. Do you agree that **broad-spectrum** antibiotics **outperform narrow-spectrum antibiotics**?
 (Note: Broad-spectrum antibiotics are able to fight most bacteria; narrow-spectrum antibiotics are dedicated to kill a particular type or a class of bacteria.)
 □ Totally agree □ Agree □ Fair □ Disagree □Totally disagree

30. Do you agree that **intravenous** antibiotics (**infusion**) are **better than oral** antibiotics?
 □ Totally agree □ Agree □ Fair □ Disagree □Totally disagree

31. Do you agree that parent can use antibiotics by himself/herself according to child's condition for treatment when they suffer from a **minor ailment**?
 □ Totally agree □ Agree □ Fair □ Disagree □Totally disagree

C. Practices

How do you use antibiotics? Please answer the following questions.

32. Is antibiotic **available at home** to medicate timely when your child is sick?

□ Always □ Often □ Sometimes □ Occasionally □ Never

33. Will you give your child **multiple antibiotics** at the same time when child is sick?
□ Always □ Often □ Sometimes □ Occasionally □ Never

34. Will you use the same antibiotics for your child **as prescribed by your doctor before** if your child has the same symptoms?
□ Always □ Often □ Sometimes □ Occasionally □ Never

35. Will you **ask your doctor to prescribe antibiotics** if he/she does not prescribe it?
□ Always □ Often □ Sometimes □ Occasionally □ Never

36. Will you **change the dosage** of antibiotics according to child's illness condition?
□ Always □ Often □ Sometimes □ Occasionally □ Never

37. Will you **change the type** of antibiotics during the process of antibiotics treatment?
□ Always □ Often □ Sometimes □ Occasionally □ Never

This is the end of our investigation.

Thanks again for your support and cooperation!
