# Supplementary material for: Understanding parental self-medication with antibiotics among parents of different nationalities: a cross-sectional study
Source: Glob Health Res Policy. 2021 Oct 25;6:42. doi: 10.1186/s41256-021-00226-y (PMC8543833; doi:10.1186/s41256-021-00226-y)
Supplement: Supplementary file 2 — Additional file 2: Questionnaire of parental self-medication with antibiotics among parents of different nationalities (Chinese Version). [file 41256_2021_226_MOESM2_ESM.pdf]

Additional file 2

不同国籍家长对儿童抗生素使用的调查

尊敬的家长朋友：

您好！近年来，家长对儿童和青少年使用抗生素在世界范围内具有高发趋势，且中外家长对抗生素使用具有不同认知和习惯，本研究希望通过对您的采访，了解您对孩子使用抗生素的情况，以及您对抗生素使用的认知、态度和行为。本调查有助于系统比较中外家长对儿童抗生素的使用，以及其抗生素知信行方面的差异，研究结果对于完善抗生素使用相关政策法规，提高群众抗生素合理使用知识具有重要意义。

问卷调查完全**自愿**并且**匿名**，仅供学术研究之用，请您放心填写。完成本问卷约需 **3-5** 分钟时间，请您抽出宝贵时间填写本问卷。

衷心感谢您的关心与支持！

✎本次调查问卷中相关用语的含义：

1. 抗生素：指用来杀死细菌的药物。它不能对抗病毒，如病毒感染引起的感冒和流感。常用抗生素包括青霉素、阿莫西林、头孢唑啉（先锋Ⅴ）、头孢拉定、头孢呋辛、头孢克肟、庆大毒素、罗红霉素、阿奇霉素等。

2. 上呼吸道感染：是自鼻腔至喉部之间的急性炎症的总称，常伴有上呼吸道肿胀和咳嗽，是最常见的自限型感染性疾病，可累及鼻、鼻窦、咽、喉和大气道。常见的症状表现为鼻塞、流鼻涕、打喷嚏、咽痛、咳嗽、低烧、头痛等。

3. 自我药疗：指为治疗自我诊断的疾病或症状而使用的药物，或为治疗慢性或复发性疾病和症状而间歇或继续使用的处方药物。

✎填表说明：

请您在相应选项的“□”内打“√”；遇到“________”请直接填写内容。

（无特别说明，每题只选一项。）

第一部分：基本情况

A. 家长：

1. 性别：

□男　　□女

2. 年龄：________岁

3. 国籍：

□中国　　　□其他亚裔　　　□欧美

4. 您是孩子的：
□父母　　　□爷爷奶奶　□其他________（请注明）

5. 文化程度：
□硕士及以上　　□本科　　□本科以下

6. 您的职业：
□公司职员　　□机关干部　　□专业技术人员
□农民　　□商人　　□个体业主　　□其他________（请注明）

7. 您的家人中是否有医务工作者：
□有　　□无

8. 您家庭的年收入情况：________（万元）

B. <u>孩子</u>：

9. 性别：
□男　　　□女

10. 年龄：________岁

11. 国籍：
□中国　　　□其他亚裔　　　□欧美

12. 班级：
□幼儿园　　□小学　□初中　　□高中

13. 是否独生子女：
□是　　□否

14. 您孩子有无医疗保险（包括居民医保和商业保险）：
□有　　□无

第二部分：家长抗生素自我药疗情况

15. 过去半年<u>**您是否（自我药疗）自行给孩子使用抗生素**</u>进行过治疗：
（注：若选择 “是”，请从第 15 题开始回答；若选择“否”，直接跳到第三部分从 21 题开始回答。）
□是　　□否

16. 过去半年您自行给孩子使用抗生素几次：________**次**；所使用抗生素的**名称**是：____；用药持续了几天：________**天**。

17. *为什么*选择给孩子自行使用抗生素？***（可多选）***
□病情较轻微，没有必要去看医生　　□之前孩子患过相同症状的疾病，有用药经验
□没有足够的空闲时间，看病时等候时间太久　　□方便　　□去医院花费较大
□其他__________（请注明）

18. 您给孩子使用抗生素是因为他/她出现了以下哪些**症状**？***（可多选）***
□流鼻涕　□鼻塞　□咳嗽　□发烧　□喉咙痛　□支气管炎
□周身疼痛　□头痛　□呕吐　□腹泻　□中耳炎　□皮肤外伤
□其他__________（请注明）

19. 您给孩子选择使用该抗生素的**依据**包括：***（可多选）***
□以前的用药经验　□家人朋友的建议　□药店工作人员推荐
□互联网知识　□药品说明书　□其他__________（请注明）

20. 您给孩子自行使用的抗生素来自以下哪些**渠道**？***（可多选）***
□以前用药的剩余　□在药店购买　□朋友赠送　□其他________（请注明）

第三部分：抗生素知信行情况

A. 知识

您对抗生素有关知识了解吗？下面请您回答抗生素的相关知识。

21. 抗生素就是**消炎药**
□对　□错　□不知道

22. 抗生素能**杀灭或抑制病毒**
□对　□错　□不知道

23. 抗生素在药店要**凭处方购买**
□对　□错　□不知道

24. 上呼吸道感染大部分是**病毒性感染**
□对　□错　□不知道

25. 经常**反复使用一种**抗生素，容易产生细菌耐药性
□对　□错　□不知道

26. 抗生素使用**剂量不足**会导致细菌耐药
□对　□错　□不知道

B. 态度

您在对孩子使用抗生素方面持什么样的态度呢？请回答以下题目。

27. 孩子患有**上呼吸道感染**时，您同意应该立即使用抗生素吗？
□ 完全同意 □同意 □一般 □不同意 □完全不同意

28. 您同意价格**昂贵的抗生素效果更好、不良反应更少**吗？
□ 完全同意 □同意 □一般 □不同意 □完全不同意

29. 您同意**广谱抗生素效果要优于窄谱抗生素**吗？
（注：广谱抗生素是指抗菌谱比较宽的药物，能够抵抗大部分细菌；窄谱抗生素是专门杀灭某一种或一类细菌的药物。）
□ 完全同意 □同意 □一般 □不同意 □完全不同意

30. 您同意**静脉注射抗生素（输液）效果优于口服抗生素**吗？
□ 完全同意 □同意 □一般 □不同意 □完全不同意

31. 孩子患有**较轻疾病**时，您同意家长可自行根据孩子病情使用抗生素进行治疗吗？
□ 完全同意 □同意 □一般 □不同意 □完全不同意

C. 行为

您在对孩子抗生素使用方面是怎样做的？请回答以下题目。

32. 您会在**家里常备抗生素**，以便孩子生病时能及时使用吗？
□一直 □经常 □有时 □偶尔 □从不

33. 孩子患病时，您会给孩子同时服用**多种抗生素**吗？
□一直 □经常 □有时 □偶尔 □从不

34. 如果您的孩子出现了跟以前相同的症状，您会**按以前医生的处方**给孩子使用抗生素吗？
□一直 □经常 □有时 □偶尔 □从不

35. 如果孩子患病去看医生，医生没有开具抗生素，您会**要求医生开抗生素**吗？
□一直 □经常 □有时 □偶尔 □从不

36. 孩子使用抗生素的过程中，您会根据孩子病情的变化自行**改变抗生素的剂量**吗？
□一直 □经常 □有时 □偶尔 □从不

37. 孩子使用抗生素的过程中，您会**更换抗生素的种类**吗？
□一直 □经常 □有时 □偶尔 □从不

调查结束，再次感谢您的支持与配合！
